# Salivaomics: New Frontiers in Studying the Relationship Between Periodontal Disease and Alzheimer’s Disease

**DOI:** 10.3390/metabo15060389

**Published:** 2025-06-10

**Authors:** Giuseppina Malcangi, Grazia Marinelli, Alessio Danilo Inchingolo, Irma Trilli, Laura Ferrante, Lucia Casamassima, Paola Nardelli, Francesco Inchingolo, Andrea Palermo, Angelo Michele Inchingolo, Gianna Dipalma

**Affiliations:** 1 Department of Interdisciplinary Medicine, University of Bari “Aldo Moro”, 70121 Bari, Italy; giuseppinamalcangi@libero.it (G.M.); graziamarinelli@live.it (G.M.); alessiodanilo.inchingolo@uniba.it (A.D.I.); irmatrilli@hotmail.com (I.T.); lauraferrante79@virgilio.it (L.F.); lucia.casamassima@uniba.it (L.C.); drnardellipaola@gmail.com (P.N.); angeloinchingolo@gmail.com (A.M.I.); gianna.dipalma@uniba.it (G.D.); 2 Departement of Experimental Medicine, University of Salento, 73100 Lecce, Italy; andrea.palermo@unisalento.it

**Keywords:** Alzheimer’s disease, metabolome, oral microbioma, saliva, periodontal disease

## Abstract

Background: This study explores the link between oral biofluids, microbial dysbiosis, and Alzheimer’s disease (AD), highlighting saliva and gingival crevicular fluid (GCF) as non-invasive diagnostic sources. AD onset and progression appear to be influenced not only by genetic and environmental factors but also by changes in the oral microbiome and related inflammatory and metabolic alterations. As global populations age, the incidence of AD is projected to rise significantly. Emerging evidence implicates the oral microbiome and salivary metabolites in neurodegenerative pathways, suggesting that oral health may mirror or influence brain pathology. Materials and Methods: A systematic review of recent multi-omics studies was performed, focusing on salivary and GCF analysis in patients with AD, those with mild cognitive impairment (MCI), and cognitively healthy individuals. Databases searched included PubMed, Web of Science, and Scopus, following PRISMA guidelines. Results: Across the 11 included studies, significant alterations were reported in both the salivary microbiome and metabolome in AD patients. Notable microbial shifts involved increased abundance of Veillonella parvula and Porphyromonas gingivalis, while key metabolites such as L-tyrosine, galactinol, and mannitol were consistently dysregulated. These biomarkers correlated with cognitive performance and systemic inflammation. Conclusions: Oral biofluids represent promising, accessible sources of biomarkers for early AD detection. Multi-omics integration reveals the oral–brain axis as a potential target for diagnosis, monitoring, and therapeutic strategies.

## 1. Introduction

**Figure 1:**
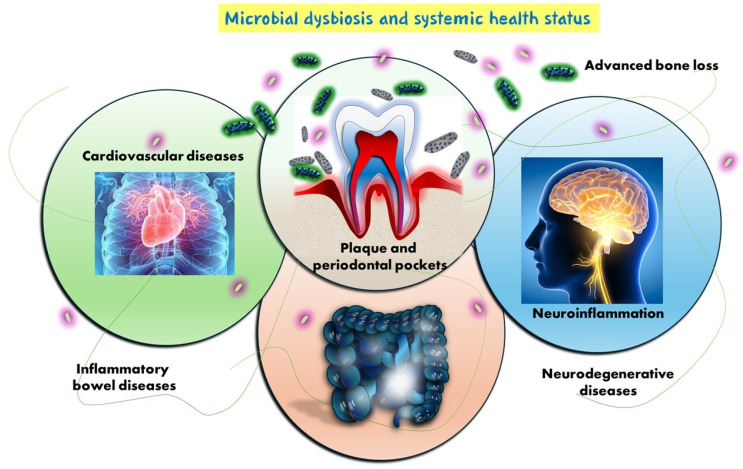
Interconnection between microbial dysbiosis and systemic health.

Alzheimer’s disease currently affects over 55 million people worldwide, a number projected to rise to 139 million by 2050, according to the World Health Organization. This dramatic increase underscores the urgent need for early, accessible, and non-invasive diagnostic tools [1,2]. The most recent data show the possibility of a ninefold increase in AD in the world’s population by 2050. This is due in part to the aging of the world’s population [3,4,5]. Scientific studies indicate that not only is AD’s onset determined by genetic and environmental factors, but also its pathogenesis may be directly influenced by microbial dysbiosis and infectious processes, and indirectly by altering metabolic, immune, and endocrine systems (Figure 1) [6,7,8,9].

**Figure 2:**
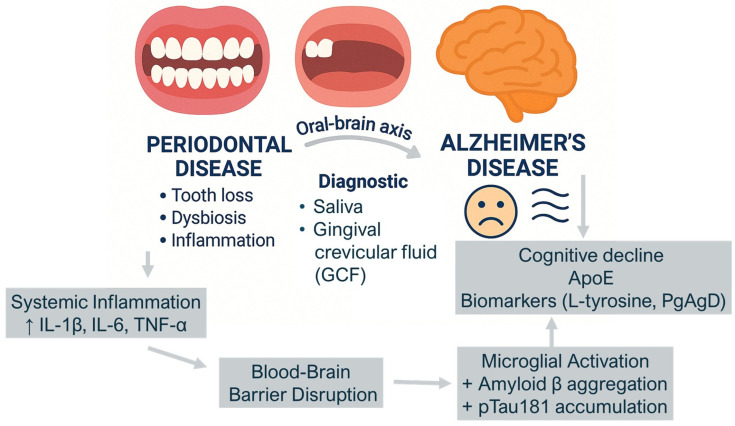
Link between periodontal disease and Alzheimer’s disease via the oral–brain axis. The arrows indicate proposed mechanisms linking periodontal inflammation to neurodegeneration: periodontal disease leads to systemic inflammation (↑ IL-1β, IL-6, TNF-α), which contributes to blood–brain barrier disruption. This promotes microglial activation and accumulation of amyloid-β and pTau181 in the brain, leading to cognitive decline. Bidirectional arrows highlight the diagnostic potential of salivary and gingival crevicular fluid biomarkers (e.g., ApoE, PgAgD), as well as the interplay between oral and neurological health.

Periodontal dysbiosis, characterized by the accumulation of pathogenic biofilm in periodontal pockets, can trigger chronic inflammation and contribute to a range of systemic conditions including cardiovascular diseases, inflammatory bowel diseases, and neurodegenerative disorders [10]. Microbial translocation and immune responses play a key role in the development of systemic inflammation, bone loss, and neuroinflammation, highlighting the oral–systemic health axis [3,4,5]. Diagnosis and prognosis of the onset and progression of AD are difficult because most studies are from advanced stages of the disease [11,12,13,14,15,16,17]. The application of metabolomics for the identification and quantification of AD metabolites, and in particular salivary metabolomics, “salivaomics”, has attracted particular interest in the scientific community due to its significant diagnostic potential [8,18,19,20,21]. The method is minimally invasive, inexpensive, and diagnostically reliable. Approximately 40% of blood proteins used in the search for physiological and pathological biomarkers (cancer, cardiovascular disease, and stroke) have been found to be present in whole saliva [22,23,24,25,26,27]. Altering the bacterial flora in saliva has been shown to significantly influence the metabolic changes and progression of Alzheimer’s [3,28,29,30,31,32]. Analysis of the salivary microbiota correlated with periodontal disease (PD) using the indicators bleeding percentage on probing (BOP), probing depth (PD), clinical attachment level (CAL), and plaque index (PI) showed a correlation with the progression of the disease, where they were more altered than those found in patients without cognitive impairment [33,34,35,36,37,38,39,40]. There have been combined studies of the gingival crevicular fluid (GCF) metabolome and subgingival microbiome in patients with AD, patients with amnestic mild cognitive impairment (aMCI), and patients in good mental and cognitive health [28,33,41,42,43,44,45,46]. Using liquid chromatography coupled to tandem mass spectrometry (LC-MS/MS) and 16S ribosomal RNA (rRNA) gene sequencing, more than 100 metabolites in GCF and more than 16 bacterial species in subgingival plaque were correlated with cognitive impairment in patients [47,48,49,50,51,52,53]. Using Data Integration Analysis for Biomarker discovery using Latent cOmponents (DIABLO), a new computational multi-omics integrative strategy that searches for common information between different types of data, more than a dozen metabolites correlated with specific bacterial strains (*Veillonella parvula, Dialister pneumosintes*, *Leptotrichia buccalis*, *Pseudoleptotrichia goodfellowii*, and *Actinomyces massiliensis*) that were predictive of the diagnosis and course of Alzheimer’s disease [54,55,56,57,58]. Data and confirmatory evidence on the oral–cerebral axis are scarce, but increasing studies show an influence of the gingival pocket microbial population (e.g., *Porphyromonas gingivalis*) on microglial activation, amyloid beta (Aβ) accumulation, and cognitive impairment [12,59,60,61,62,63,64,65,66]. Recent data suggest a dual mechanism by which the oral microbiota and their metabolites contribute to brain dysfunction: directly, by crossing the blood–brain barrier and inducing local neuroinflammatory responses, and indirectly, by promoting systemic inflammation via chronic periodontal disease, which elevates circulating pro-inflammatory cytokines such as interleukin-1β (IL-1β), tumor necrosis factor-α (TNF-α), and interleukin-6 (IL-6), all implicated in the pathogenesis of Alzheimer’s disease [54,55,67,68,69,70,71,72,73]. *Veilonella parvula*, a Firmicutes bacterium associated with periodontitis, is an opportunistic intracranial pathogen. Through the bloodstream, *V. parvula* can enter the brain, stimulate microglia, cause inflammation of nerve cells, and ultimately lead to AD and cognitive impairment [74,75,76,77,78,79,80]. Tooth loss caused by PD, as well as poor oral hygiene in people with Alzheimer’s or dementia, leads to impaired chewing, which not only makes eating more difficult with less food, but also reduces cerebral blood flow (Figure 2) [81,82,83,84,85,86].

Chronic inflammation and dysbiosis associated with periodontal disease may contribute to neurodegenerative processes seen in Alzheimer’s disease. Saliva and gingival crevicular fluid are emerging as promising non-invasive diagnostic sources for identifying biomarkers relevant to both oral and neurodegenerative conditions.

Reduced chewing activity has been correlated with reduced acetylcholine levels and the number of pyramidal cells involved in the hippocampus, resulting in impaired short- and long-term memory, spatial memory, learning, and emotional co-occurrence [87,88,89,90,91,92]. For example, studies have shown that the presence of toothlessness can have a detrimental effect on the course of neurodegenerative diseases such as AD [93,94]. Given that approximately 73% of salivary proteins are not present in plasma, whereas 40% of plasma proteins are present in saliva, our research aims to highlight how salivary metabolomics and proteomics can offer unique opportunities to detect and use salivary proteomic markers for the diagnosis of neurodegenerative diseases such as Alzheimer’s, as well as other diseases that are not only oral but also systemic, with the added advantage of multiple sampling and a non-invasive method [22]. Despite growing evidence linking periodontal dysbiosis, salivary biomarkers, and Alzheimer’s disease, current studies remain limited by heterogeneity in methodology, small sample sizes, and a lack of longitudinal data. Furthermore, most existing research focuses on either the microbiome or metabolome in isolation, without a fully integrated multi-omics approach. Few studies concurrently assess both gingival crevicular fluid and saliva using comprehensive omics technologies in well-characterized AD and MCI populations. Therefore, there is a critical need for systematic reviews that synthesize findings across multi-omics studies to clarify the diagnostic and mechanistic potential of oral biofluids in neurodegenerative disease. This review aims to address this gap by evaluating the current literature on the salivary and subgingival microbiome and metabolome in relation to cognitive decline.

The aim of this comprehensive review is to evaluate current multi-omics evidence on the salivary and gingival crevicular fluid (GCF) microbiome and metabolome in Alzheimer’s disease, to assess their potential as non-invasive diagnostic and mechanistic biomarkers.

## 2. Materials and Methods

### 2.1. Search Strategy

This systematic review followed the principles of methodological rigor recommended by PRISMA, registered in PROSPERO with ID: 1042244. A thorough electronic search was carried out using three major scientific databases—PubMed, Web of Science, and Scopus—to identify relevant literature published between 2015 and 2025. This time frame was selected to capture the most recent advances in multi-omics technologies and analytical approaches—such as salivary metabolomics, 16S rRNA sequencing, and LC-MS/MS—applied to the study of the oral–brain axis. Earlier studies often lacked these tools or did not focus on the mechanistic links now central to the research question.

The search strategy was constructed by combining controlled vocabulary and keywords related to salivary biomarkers and systemic conditions. Specifically, the Boolean combination used was (metabolome OR metabolite OR saliva OR salivome) AND (“Alzheimer’s Disease” OR “periodontal disease”). Relevant publications from the period 2015 to 2025 were identified by systematically searching three electronic databases: PubMed, Web of Science, and Scopus. Although the search strategy did not explicitly include the terms “biomarker,” “salivary biomarker,” or “periodontitis,” the use of broader terms such as “saliva,” “salivome,” and “periodontal disease” allowed for the inclusion of studies addressing these aspects. However, the absence of certain specific keywords may have limited the retrieval of additional relevant literature and should be considered when interpreting the comprehensiveness of the search. The complete search strings for each database, along with the number of records retrieved, are provided in Table 1.

### 2.2. Eligibility Criteria and PICO Framework and Research Question

Studies were eligible for inclusion if they met the following criteria:

- Population: Human subjects of any age diagnosed with Alzheimer’s disease (AD) or mild cognitive impairment (MCI), or cognitively healthy controls, with or without a diagnosis of periodontal disease (PD).
- Intervention/Exposure: Assessment of salivary metabolomic or microbiome profiles, including techniques such as LC-MS/MS, GC-MS, NMR, and 16S rRNA sequencing. Studies analyzing gingival crevicular fluid (GCF) were also considered if saliva was included.
- Comparison: Studies comparing subjects with AD versus healthy controls, or patients with periodontitis versus those without, or studies examining within-group correlations (e.g., between salivary markers and cognitive scores).
- Outcomes: Diagnostic or prognostic relevance of salivary biomarkers, correlation with clinical measures (e.g., MMSE, MoCA, periodontal indices), or characterization of oral microbial/metabolomic signatures in systemic disease contexts.
- Study Design: Observational studies including cross-sectional, case–control, and cohort designs, as well as randomized controlled trials (RCTs) where applicable.
- Language and Access: Published in English and available in full text.

Studies were excluded if they

- Were conducted using in vitro or animal models;
- Were narrative or systematic reviews, editorials, case reports, or conference abstracts lacking sufficient methodological detail;
- Did not include salivary (or GCF) analysis relevant to AD or PD;
- Focused exclusively on unrelated conditions without addressing oral–systemic interactions.

These criteria aimed to ensure the inclusion of clinically relevant, methodologically sound studies that explore the interface between oral health and neurodegenerative disease through salivary diagnostics.

The research question was developed using the PICO framework to provide a structured approach to study selection and synthesis:

Population (P): Human participants with Alzheimer’s disease, mild cognitive impairment, or periodontal disease; may include cognitively healthy controls as comparators.

Interest (I): Salivary metabolomics and microbiome profiles, including inflammatory mediators, bacterial load (e.g., *P. gingivalis*), and molecular biomarkers potentially linked to systemic conditions.

Comparison (C): Comparisons across disease stages, presence vs. absence of periodontal disease, or differing biomarker levels among clinical subgroups.

Outcome (O): Evidence of associations between salivary biomarkers and clinical parameters (e.g., cognitive decline, periodontal status), as well as their potential utility in early detection or monitoring of neurodegenerative and inflammatory oral conditions.

The objective of this review was to identify and critically appraise current evidence on salivary metabolomic and microbiome signatures associated with Alzheimer’s disease and/or periodontal disease, focusing on their potential use as non-invasive biomarkers and their mechanistic relevance within the oral–brain axis.

### 2.3. Data Extraction and Analysis

Title and abstract screening was independently conducted by two reviewers (L.C. and L.F.), while full-text screening was carried out by three reviewers (L.C., L.F., and I.T.). In cases of disagreement during the eligibility assessment, a subject expert (P.N.) was consulted for a resolution. Data extraction from the included studies was independently performed by all four reviewers (L.C., L.F., I.T., and P.N.) using a standardized form developed in Microsoft Excel. Collected variables included study design, population characteristics, analytical techniques, metabolomic findings, disease outcomes, and follow-up duration (if applicable). Any inconsistencies in data extraction were discussed and resolved through consensus, with P.N. acting as the third-party adjudicator when needed. Descriptive statistics were used to summarize the included studies. Due to heterogeneity in study designs and outcomes, a meta-analysis was not feasible; instead, a narrative synthesis was conducted.

## 3. Results

### 3.1. Study Selection and Characteristics

**Figure 3:**
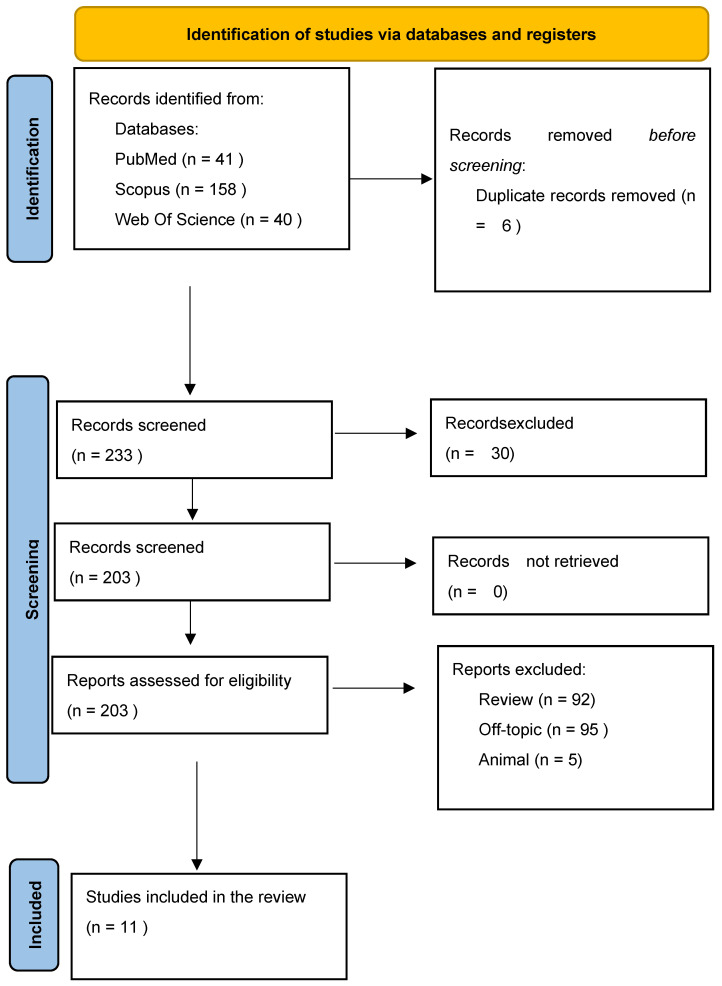
PRISMA 2020 flow diagram. (“This PRISMA flow diagram was adapted from the PRISMA 2020 template (http://www.prisma-statement.org) under a CC BY 4.0 license.”) (accessed on 30 March 2025).

A total of 239 records were initially retrieved through database searches: 41 from PubMed, 40 from Web of Science, and 158 from Scopus. After removing 6 duplicates, 233 records remained for title and abstract screening. Of these, 30 studies were excluded for the following reasons: lack of salivary analysis, use of in vitro models, non-relevant outcomes, or insufficient methodological detail. After this step, 192 records were excluded because 5 studies were on animal models, 92 were reviews, and 95 were off topic. Ultimately, 11 studies met the inclusion criteria and were incorporated into the qualitative analysis. The selection process is illustrated in Figure 3, and the detailed characteristics of the included studies are presented in Table 1.

### 3.2. Synthesis of Findings Across Studies

Among the studies included, several consistent patterns emerged. François et al. (2024) and François et al. (2021) identified salivary biomarkers such as Stratifin (14-3-3σ) and L-tyrosine, which were significantly correlated with plasma pTau181, indicating potential for early, non-invasive diagnosis of AD [95,96]. Both studies also reported significant changes in salivary microbiota composition in AD and MCI patients. Issilbayeva et al. (2024) found that AD patients exhibited increased oral microbial diversity, with a notable decrease in taxa like *Haemophilus parainfluenzae* and *Prevotella* (*Prevotella melaninogenica, Prevotella histicola*, and *Prevotella intermedia*) [97]. Similarly, Qiu et al. (2024) identified specific bacterial species (e.g., *Veillonella parvula, Dialister pneumosintes*) and metabolites in gingival crevicular fluid that were strongly associated with cognitive impairment and periodontal severity, achieving high discriminatory power (AUC > 0.98) [33]. Sansores-España et al. (2022) provided evidence linking severe periodontitis in AD patients with increased levels of IL-1β, IL-9, IL-22, *P. gingivalis*, and ApoE-ε4 in GCF [98]. Notably, ApoE-ε4 showed a diagnostic AUC of 84.9% for AD, underscoring the biomarker potential of GCF. Guo et al. (2023) and Na et al. (2024) confirmed that AD patients had a distinct oral microbiome enriched in pathogenic bacteria like *P. gingivalis* and *F. alocis*, while beneficial species were reduced [28,99]. Hamdi et al. (2024) highlighted a downregulation of PgAgD in AD with advanced periodontitis, further reinforcing the oral–neuro axis [100]. Across all studies, the most consistent findings were (1) shifts in oral microbiota composition in AD patients, (2) increased inflammatory cytokines in GCF, and (3) the detection of salivary and GCF biomarkers correlated with both periodontal disease and cognitive decline. These data collectively support the potential of oral biofluids as sources of diagnostic biomarkers for AD.

### 3.3. Risk of Bias Assessment

The risk of bias in the included studies was evaluated using the ROBINS-I tool, which assesses non-randomized studies across multiple methodological domains. Overall, the studies were found to carry a serious risk of bias, primarily due to confounding factors and potential inaccuracies in outcome measurement. Confounding remains a major limitation in observational research, as unmeasured or insufficiently controlled variables—such as patient demographics, disease severity, genetic predispositions, and lifestyle factors—may influence both exposure and outcomes. Although several studies employed strategies to adjust for potential confounders, the risk of residual confounding cannot be excluded. Selection bias was generally rated as moderate. While inclusion and exclusion criteria were typically well defined, many studies relied on convenience sampling rather than representative, population-based recruitment, limiting generalizability. The classification of exposures, including assessments of periodontal status, salivary biomarkers, and oral microbiota profiles, was considered at low risk of bias, as most studies used validated and standardized protocols. Given the observational nature of all included studies, deviations from intended interventions were not applicable and thus not a concern. Missing data was not a significant issue in most studies, with acceptable follow-up rates and generally complete datasets reported. However, a moderate risk of bias was frequently identified in the measurement of outcomes. Despite the use of validated tools for both cognitive and periodontal assessment, inconsistencies in examiner calibration and laboratory procedures may have introduced measurement error.

Selective reporting bias was typically assessed as low, as most studies reported their prespecified outcomes. Nevertheless, in a few cases, there was a lack of clarity regarding the reporting of secondary or exploratory analyses. In summary, although the collective evidence highlights relevant associations between oral health parameters and Alzheimer’s disease, these findings must be interpreted with caution. The serious risk of bias observed across several domains suggests that conclusions drawn from these studies should be regarded as exploratory and hypothesis-generating rather than conclusive. Future research should prioritize longitudinal designs, rigorous confounder control, and the application of standardized methodologies to validate and expand upon these preliminary findings. A detailed summary of the risk of bias assessment for each study is presented in Table 2.

### 3.4. Summary of Findings, Evidence Certainty (GRADE), Publication Bias, Heterogeneity, and Meta-Analysis Considerations

#### 3.4.1. GRADE Assessment

A formal GRADE evaluation was not performed due to substantial heterogeneity in study designs, populations, biomarkers, and outcome measures. Nevertheless, the overall certainty of evidence can be informally rated as low to moderate, primarily due to the serious risk of bias, methodological inconsistencies, use of surrogate outcomes, and small sample sizes. These limitations underscore the need for future well-designed longitudinal or interventional studies.

#### 3.4.2. Publication Bias

A formal assessment of publication bias (e.g., funnel plot, Egger’s test) was not feasible due to the absence of a meta-analysis and the limited number of included studies, which would not allow for a reliable statistical evaluation. However, the predominance of positive findings, especially from small or pilot studies, raises the possibility of selective reporting and publication bias.

#### 3.4.3. Meta-Analysis

Meta-analysis was not performed due to substantial heterogeneity in study design, biological samples, biomarkers assessed, definitions of periodontal and cognitive status, and lack of standardized outcome measures. These differences prevented data pooling and quantitative synthesis.

#### 3.4.4. Heterogeneity

High heterogeneity was observed across studies in terms of participant characteristics, sample collection methods, analytical platforms, and outcome definitions. This variability further justifies the qualitative nature of the synthesis and highlights the need for standardized protocols in future research.

## 4. Discussion

Recent years have seen a growing interest in the role of the oral microbiome and oral biofluids in neurodegenerative diseases, particularly AD [101,102,103,104,105]. The studies examined here focus on saliva, GCF, and subgingival plaque, using increasingly sophisticated multi-omics tools to uncover microbial, metabolic, proteomic, and transcriptomic changes associated with cognitive decline [106,107,108,109,110,111]. While each study explores different aspects of this complex relationship, taken together, they provide a coherent narrative of the oral cavity as a potential mirror—and perhaps driver—of neurodegenerative pathology [111]. Several studies reported significant differences in salivary microbiome composition and inflammatory profiles among participants when comparing individuals with Alzheimer’s disease (AD), those with mild cognitive impairment (MCI), and cognitively normal (CHN) controls. For instance, patients with AD showed a higher abundance of *P. gingivalis* and elevated pro-inflammatory cytokines compared to both the MCI and CN groups, while the MCI group exhibited intermediate levels, suggesting a potential continuum in disease progression. Among the most comprehensive are the two multi-omics studies by François et al. (2021) and François et al. (2024), which provide a detailed picture of salivary changes in AD [95,96]. Their integrative analyses revealed significant shifts in the salivary proteome, metabolome, and microbiome, highlighting proteins like Stratifin and metabolites such as L-tyrosine and 3-chlorotyrosine, both linked to oxidative stress and inflammation [95]. Notably, the diversity of the salivary microbiome was reduced in AD patients, with a depletion of commensal bacteria and a rise in potential pathogens [95]. The 2024 follow-up study confirmed these findings in a different cohort and added further depth by correlating oral changes with plasma biomarkers such as pTau181 [95]. These results suggest that saliva may not only reflect peripheral markers of AD but could also capture central neuropathological changes [95].

While François et al. focused heavily on the interplay of salivary components, Yang et al. (2023) emphasized the relationship between the salivary metabolome and specific microbial taxa [3]. Their identification of four discriminatory metabolites—particularly taurine and 3-hydroxybutyric acid—demonstrates that oral metabolic changes may be tightly coupled with microbial dysbiosis. The novelty of their approach lies in highlighting how specific bacteria may contribute to the accumulation of neurotoxic compounds, suggesting a potentially active role of oral microbes in systemic disease mechanisms [3].

This idea is echoed in the work of Sansores et al., who examined inflammatory cytokines in both saliva and GCF in patients with AD and periodontitis [98]. They reported elevated levels of IL-1β, TNF-α, and IL-6 in the GCF of cognitively impaired patients, especially those carrying the ApoE-ε4 allele. These inflammatory profiles were associated with higher levels of *P. gingivalis*, reinforcing the hypothesis that local oral inflammation—when combined with genetic susceptibility—may contribute to or exacerbate neuroinflammation [99].

The study by Qiu et al. also places the spotlight on GCF, but from a metabolomic perspective. They found that changes in bacterial species, particularly Veillonella parvula and Dialister pneumosintes, were associated with alterations in metabolites such as galactinol and mannitol. This reinforces the idea that GCF, though less frequently studied than saliva, may offer a rich source of information about both microbial and host-derived metabolic changes related to cognitive decline [33].

The role of subgingival microbiota is explored in more detail by Guo et al. (2023) and Na et al. (2024) [28,99]. Guo et al. observed reduced microbial diversity in the subgingival plaque of AD patients, with a dominance of pathogens like *P. gingivalis* and *F. alocis*, even in the absence of clinical periodontal disease [28]. This suggests that microbial imbalance in the oral cavity may precede or parallel neurodegenerative changes [28]. Na et al. expanded on this idea by showing that when AD coexists with periodontitis, the microbial ecosystem becomes even more unstable, with a unique pro-inflammatory profile not seen in periodontitis alone [99]. This supports a bidirectional relationship in which AD may alter local immunity, thereby reshaping the microbial landscape of the oral cavity [112].

In contrast to studies reporting reduced microbial diversity in AD, Issilbayeva et al. found the opposite: an increased richness of the oral microbiome in cognitively impaired individuals [97]. This highlights the variability in microbiome findings across studies, which may reflect differences in sampling techniques, populations, or analytical methods. However, their observation of phylum-level shifts (increased Firmicutes and decreased Bacteroidetes) and species-level reductions in Haemophilus parainfluenzae and Actinomyces oris points to a clear microbial imbalance [97]. Moreover, their functional pathway analysis showed disruptions in amino acid biosynthesis, reinforcing the idea that microbiota may affect not only local but also systemic metabolic pathways [97].

Going beyond microbial presence, Hamdi et al. (2024) explored microbial gene expression, focusing on agmatine deiminase (PgAgD) from *P. gingivalis* [100]. They found that its expression was significantly lower in the saliva of AD patients and inversely correlated with cognitive scores [113]. This transcriptomic approach represents an important shift from merely cataloguing microbial taxa to understanding their actual functional activity, and suggests that bacterial gene expression patterns could serve as novel early markers of cognitive decline [113].

One of the few longitudinal studies in this field, conducted by Chen et al. (2024), tracked changes in the subgingival microbiome across the spectrum from healthy aging to MCI and AD [74]. They reported a gradual loss of microbial richness, with a steady rise in *P. gingivalis* and a decline in beneficial genera such as Capnocytophaga and Granulicatella. Crucially, functional pathways related to lipid metabolism and immune modulation were active only in MCI and AD, suggesting that changes in microbial function precede or accompany clinical cognitive decline [74].

Finally, Ide et al. conducted a six-month observational study to investigate the association between periodontitis and cognitive decline in individuals with Alzheimer’s disease (AD) [91]. While the presence of periodontitis at baseline was not associated with initial cognitive performance, it was significantly linked to a six-fold increase in the rate of cognitive decline, as measured by the ADAS-Cog. This association remained significant even after adjusting for age, sex, and baseline cognitive function. Moreover, patients with periodontitis exhibited elevated levels of systemic pro-inflammatory markers and decreased levels of anti-inflammatory cytokines, suggesting that systemic inflammation may mediate the observed cognitive deterioration. These findings support the hypothesis that periodontitis may represent a modifiable risk factor for accelerated cognitive decline in AD.

Taken together, the studies included in this review reveal a complex yet consistent pattern: the oral cavity, far from being an isolated system, is intricately connected to brain health. Despite methodological variability and differences in specific findings, there is a general consensus that a dysbiotic oral microbiome, along with altered metabolic and immune profiles, may not only reflect but also contribute to neurodegenerative disease processes. Saliva and gingival crevicular fluid (GCF), in particular, have emerged as promising non-invasive sources of biomarkers, capable of capturing shifts in microbial composition, host immune response, and microbial functional activity.

In conclusion, the convergence of microbial, metabolic, and inflammatory signatures in oral biofluids presents a compelling rationale for their inclusion in future diagnostic and monitoring strategies for Alzheimer’s disease. The integration of multi-omics approaches across the reviewed studies represents a significant advancement in elucidating the oral–systemic axis in neurodegeneration, and opens new avenues for early detection, risk stratification, and potentially even therapeutic intervention. Table 3 summarizes the key findings from the included studies.

The main outcomes and methodological assessments of the included studies are reported in Table 4. Summary of key findings from included studies, highlighting the overall risk of bias and the domains most frequently affected.

## 5. Limitation

There are various restrictions on this review. There was minimal comparability and no quantitative meta-analysis due to the variability in study designs, salivary biomarkers examined, and methodologies. Numerous included studies had small sample sizes and were cross-sectional, which limited the ability to draw conclusions about causality and decreased statistical power. Furthermore, methodological bias might have been introduced due to a lack of established procedures for saliva collection and multi-omics analysis. The results’ generalizability is further limited by the lack of longitudinal data and uneven confounder adjustment for factors like age, comorbidities, and dental hygiene habits. Standardized, prospective designs with larger cohorts and improved confounding factor control should be used in future research.

## 6. Conclusions

The collective evidence from the included studies demonstrates that oral biofluids, particularly saliva and GCF, offer a viable, non-invasive platform for identifying biomarkers associated with AD. The integrated multi-omics analyses reveal consistent alterations in microbial composition, metabolic profiles, and local inflammatory responses that correlate closely with cognitive impairment and disease progression. Specifically, the data indicates a reproducible pattern of microbial dysbiosis characterized by an increase in pathogenic taxa such as *Porphyromonas gingivalis* and *Fretibacterium alocis*, alongside a reduction in beneficial commensals. Concurrently, elevated pro-inflammatory cytokines in GCF and salivary biomarkers such as Stratifin and L-tyrosine are significantly associated with established plasma markers of AD neuropathology (e.g., pTau181). These findings collectively support the hypothesis that oral microbial and molecular signatures not only reflect peripheral disease processes but may also participate in mechanisms underlying neurodegeneration. Importantly, several studies highlight the relationship between periodontal disease severity, oral microbial changes, and accelerated cognitive decline, suggesting a bidirectional oral–brain axis that warrants further investigation. While these cross-sectional observations provide compelling correlations, the limited longitudinal data and variability in methodologies preclude definitive causal inferences at this stage. In conclusion, the evidence generated by the reviewed research directly addresses the research question by substantiating the diagnostic and prognostic potential of oral biofluid-derived biomarkers in AD. The convergence of microbial, metabolic, and inflammatory signatures in saliva and GCF underscores their relevance for early detection and disease monitoring. Future work employing standardized, longitudinal designs with larger cohorts is essential to validate these biomarkers and clarify their role in AD pathophysiology and clinical application.

## Figures and Tables

**Table 1:** Search strings and number of records retrieved from each database.

Database	Search String	Number of Record Retrieved
Pubmed	(metabolome OR metabolite OR saliva OR salivome) AND (“Alzheimer disease” OR “periodontal disease”)	41
Web of science	(metabolome OR metabolite OR saliva OR salivome) AND (“Alzheimer disease” OR “periodontal disease”)	40
Scopus	(metabolome OR metabolite OR saliva OR salivome) AND (“Alzheimer disease” OR “periodontal disease”)	158

**Table 2:** Articles included in the discussion section.

References	Type of Study	N. of Patients	Aim of the Study	Outcomes
François et al. (2024) [95]	Cross-sectional, observational	80 participants: 40 CHC, 20 MCI, 20 AD	- Saliva and plasma samples. - Proteomics, metabolomics, and microbiome (16S rRNA) analysis. - Correlation with known plasma biomarkers including pTau181.	- Stratifin (14-3-3σ) and L-tyrosine as top saliva biomarkers highly correlated with plasma pTau181. - Salivary microbiome changes observed (↓ *Lautropia mirabilis* in AD/MCI).
François et al. (2021) [96]	Observational, cross-sectional pilot study	80 participants: 40 CHC, 20 with MCI, 20 with AD	- Saliva and blood samples collected. - Multi-omics analysis via untargeted mass spectrometry-based proteomics and metabolomics.	- Identified 15 significantly dysregulated metabolic pathways in AD and MCI. - Key changes included dysregulation in glycolysis, citric acid cycle, tyrosine/phenylalanine metabolism, and immune-related proteins. - Saliva shown to be a viable, stress-free medium for early AD biomarker discovery.
Issilbayeva et al. (2024) [97]	Case–control study	135 participants: 64 with AD, 71 CHC	- Oral swabs from multiple intraoral sites. - 16S rRNA gene sequencing. - Clinical periodontal evaluation. - Metabolic pathway analysis.	- AD group showed increased oral microbiome diversity. - Reduced abundance of specific taxa (*Haemophilus parainfluenzae*, *Prevotella*, *Actinomyces*) in AD. - No direct association between AD and periodontitis. - Significant differences in metabolic pathways (e.g., histidine, lysine biosynthesis). - Identified microbiome markers with predictive power for AD (e.g., *Anaerostipes*, *Lactobacillus*).
Sansores-España et al. (2022) [98]	Clinical observational pilot study	30 participants: CHC, periodontitis without AD, and periodontitis with AD	- Periodontal examination and Montreal Cognitive Assessment (MoCA) cognitive test. - Collection of GCF and subgingival microbiota. - Analysis of cytokines and *P. gingivalis*.	- AD patients had more severe periodontitis (stage IV in 80%). - Higher levels of pro-inflammatory cytokines (IL-1β, IL-9, IL-22), bacterial load of *P. gingivalis*, and ApoE-ε4 in GCF. - Negative correlation between these biomarkers and cognitive performance (MoCA score). - ApoE-ε4 detection in GCF had an Area Under the Curve (AUC) of 84.9%, suggesting diagnostic potential for AD.
Qiu et al. (2024) [33]	Cross-sectional, observational study	96 participants: 32 CHC, 32 MCI, and 32 AD	- Collection of subgingival plaque and GCF. - 16S rRNA gene sequencing of microbiota.	- Identified 16 bacterial species and 165 GCF metabolites associated with cognitive decline. - *Veillonella parvula* and *Dialister pneumosintes* were enriched in AD and correlated with poor periodontal and cognitive scores. - Certain metabolites (e.g., galactinol, D-mannitol) had high discriminative power (AUC > 0.98) for distinguishing AD from CN/aMCI.
Guo et al., 2023 [28]	Observational (cross-sectional) study	60 participants (33 with AD, 27 CHC)	To investigate oral microbiomes of Alzheimer’s disease patients and controls.	AD patients had an altered oral microbiome composition, with increased pathogenic bacteria (*P. gingivalis*, *F. alocis*) and reduced beneficial bacteria (*R. mucilaginosa*, *C. matruchotii*). Changes were more pronounced in subgingival plaque and seemed independent of oral hygiene status.
Ide et al., 2016 [91]	Observational cohort (6 months)	60 participants	To investigate the correlation between periodontitis and AD.	Periodontitis is associated with significantly faster cognitive decline and heightened systemic inflammation in AD patients.
Na et al., 2024 [99]	Cross-sectional observational study	43 participants (21 with AD + PD; 22 with PD only)	To investigate the influence between AD and periodontitis.	AD influences oral microbiota and may worsen periodontal condition.
Hamdi et al., 2024 [100]	Observational, cross-sectional	54 participants (27 AD patients, 27 CHC)	To investigate the dysregulation of *Porphyromonas gingivalis* agmatine deiminase (PgAgD) expression in Alzheimer’s disease and its correlation with periodontitis and cognitive decline.	Correlation between decreased PgAgD expression and lower Mini-Mental State Examination (MMSE) scores.
Chen et al. (2024) [74]	Cross-sectional study	165 older adults	To explore differences in subgingival microbiota composition and function across cognitive states.	Decreased microbial richness with cognitive decline; specific bacterial signatures associated with cognitive level.
Yang et al. (2023) [3]	Observational case–control study	79 participants (60 for saliva analysis)	To investigate the correlation between periodontal status and identify salivary metabolic biomarkers in AD.	AD linked with worse periodontal status; four salivary biomarkers identified.

Abbeviations in the table: AD (Alzheimer’s disease), AUC (Area Under the Curve), CHC (cognitively healthy controls), GCF (gingival crevicular fluid), MCI (mild cognitive impairment), MMSE (Mini-Mental State Examination), MoCA (Montreal Cognitive Assessment), PD (periodontal disease), PgAgD (*Porphyromonas gingivalis* agmatine deiminase), rRNA (ribosomal ribonucleic acid). ↓ means Reduction.

**Table 3:** The risk of bias assessment is summarized for each study across the seven ROBINS-I domains.

Study	Confounding	Selection of Participants	Classification of Interventions	Deviations from Intended Interventions	Missing Data	Measurement of Outcomes	Selection of Reported Results	Overall Risk
François et al. (2024) [95]	Moderate risk	Low risk	Low risk	Low risk	Low risk	Moderate risk	Low risk	Moderate risk
François et al. (2021) [96]	Low risk	Low risk	Low risk	Low risk	Low risk	Moderate risk	Low risk	Low risk
Issilbayeva et al. (2024) [97]	Moderate risk	Moderate risk	Low risk	Low risk	Low risk	Moderate risk	Low risk	Moderate risk
Sansores-España et al. (2022) [98]	Serious risk	Moderate risk	Low risk	Low risk	Low risk	Moderate risk	Low risk	High risk
Qiu et al. (2024) [33]	Low risk	Low risk	Low risk	Low risk	Low risk	Low risk	Low risk	Low risk
Guo et al. (2023) [28]	Low risk	Low risk	Low risk	Low risk	Low risk	Moderate risk	Low risk	Low risk
Ide et al. (2016) [91]	Low risk	Low risk	Low risk	Low risk	Low risk	Moderate risk	Low risk	Moderate risk
Na et al. (2024) [99]	Low risk	Low risk	Low risk	Low risk	Low risk	Moderate risk	Low risk	Moderate risk
Hamdi et al. (2024) [100]	Low risk	Low risk	Low risk	Low risk	Low risk	Low risk	Low risk	Low risk
Chen et al. (2024) [74]	Low risk	Low risk	Low risk	Low risk	Low risk	Low risk	Low risk	Low risk
Yang et al. (2024) [3]	Moderate risk	Low risk	Low risk	Low risk	Low risk	Moderate risk	Low risk	Moderate risk

**Table 4:** Summary of key findings from included studies.

Key Finding	Inference/Implication
↓ Oral microbial diversity in AD ↑ *P. gingivalis*, *F. alocis*, ↑ *Firmicutes*	Oral dysbiosis is associated with cognitive decline and may precede clinical signs of Alzheimer’s Disease (AD)
↑ Salivary metabolites (e.g., L-tyrosine, 3-chlorotyrosine, taurine, 3-hydroxybutyric acid)	Oral metabolic changes reflect oxidative stress and inflammation, potentially linked to neurodegenerative processes
↑ Inflammatory cytokines (IL-1β, IL-6, TNF-α) in saliva and GCF	Local oral inflammation may contribute to systemic neuroinflammation, particularly in genetically susceptible individuals (e.g., ApoE-ε4 carriers)
↓ Expression of beneficial microbial genes (e.g., PgAgD)	Microbial functional changes suggest an active role of the oral microbiome in AD pathophysiology
Coexistence of AD and periodontitis = ↑ Inflammatory profile + ↑ Cognitive decline	Periodontal disease may be a modifiable risk factor for the progression of Alzheimer’s Disease
Gradual microbial and metabolic changes from healthy aging → MCI → AD	Oral biofluids could serve as early biomarkers for the diagnosis and monitoring of cognitive decline

## Data Availability

No new data were created or analyzed in this study. Data sharing is not applicable to this article.

## Abbreviations

The following abbreviations are used in this manuscript:

Aβ	Amyloid beta
AD	Alzheimer’s disease
AUC	Area Under the Curve
aMCI	Amnestic mild cognitive impairment
BOP	Bleeding on probing
CAL CHC	Clinical attachment level Cognitively healthy control
DIABLO	Data Integration Analysis for Biomarker discovery using Latent cOmponents
GCF	Gingival crevicular fluid
IL-1	Interleukin-1
IL-6	Interleukin-6
LC-MS/MS	Liquid chromatography coupled to tandem mass spectrometry
MoCA	Montreal Cognitive Assessment
PD	Periodontal disease
PgAgD	Agmatine deiminase from Porphyromonas gingivalis
PI	Plaque index
pTau181	Phosphorylated Tau protein at threonine 181
rRNA	Ribosomal ribonucleic acid
TNF-α	Tumor necrosis factor-alpha
